# Risk Factors for Adverse Outcomes in Connective Tissue Disease-Associated Pulmonary Hypertension

**DOI:** 10.31083/RCM26877

**Published:** 2025-03-17

**Authors:** Gayane Matusov, Maryam Shams, Karim Ibrahim, Areg Hovsepyan, Yuri Matusov

**Affiliations:** ^1^Department of Internal Medicine, Scripps Clinic, La Jolla, CA 92037, USA; ^2^Department of Internal Medicine, Sutter Roseville Medical Center, Roseville, CA 95661, USA; ^3^Department of Internal Medicine, Cedars-Sinai Medical Center, Los Angeles, CA 90048, USA; ^4^Department of Hospital Medicine, Adventist Health Simi Valley, Simi Valley, CA 93065, USA; ^5^Department of Medicine, Division of Pulmonary & Critical Care Medicine, Cedars-Sinai Medical Center, Los Angeles, CA 90048, USA

**Keywords:** pulmonary hypertension, connective tissue disease, autoimmune disease, scleroderma, systemic lupus erythematosus

## Abstract

Pulmonary hypertension (PH) is a rare, life-threatening condition that can be associated with connective tissue disease (CTD). The incidence and prevalence of PH in CTD varies by disease, whereby certain disease manifestations are particularly associated with PH; nonetheless, once present, PH is almost uniformly a major driver of adverse outcomes. In this paper, the authors review the published literature on major CTDs, including systemic sclerosis and systemic lupus erythematosus, and summarize the risk factors for developing PH in each disease and risk factors for adverse outcomes and mortality among patients with CTD-PH. This review highlights the need for early diagnosis of PH in CTD and the impact of PH overlap syndromes on patient outcomes, providing the practicing clinician with a practical summary of CTD-PH.

## 1. Introduction

Pulmonary hypertension (PH) is a rare, life-threatening condition which can be associated with connective tissue disease. It is diagnosed by right heart catheterization, with a mean pulmonary artery (PA) pressure of greater than 20 mmHg with pulmonary vascular resistance (PVR) greater than 2 Wood units (WU), as established by the World Symposium on Pulmonary Hypertension (WSPH) [1]. Patients are classified as having precapillary PH if pulmonary capillary wedge pressure (PCWP) is ≤15 mmHg, which includes patients with pulmonary arterial hypertension (PAH) (WSPH group 1), PH associated with chronic lung disease (WSPH group 3) and chronic thromboembolic pulmonary hypertension (CTEPH, WSPH group 4) [1].

Connective tissue disease (CTD) associated with PH is usually classified under WSPH group 1, with CTD-PAH having a variable prevalence of between 15–25% in PH registries [2, 3]. These patients’ outcomes, including survival, may be worse than that of patients with idiopathic PAH [4]. However, there is an extraordinary complexity in these patients’ presentations, overlap syndromes between different CTDs, overlap syndromes between different classes of PH, and particular patient-related characteristics which can significantly affect patients’ outcomes. This paper presents a scoping review of the literature published on factors associated with adverse outcomes in patients who have CTD-associated PH, highlighting specific issues relevant for clinicians caring for these patients. A summary of known risk factors associated with the development of PH in CTD and known risk factors associated with mortality in CTD-PH is provided in Table 1 (Ref. [5, 6, 7, 8, 9, 10, 11, 12, 13, 14, 15, 16, 17, 18, 19, 20, 21, 22, 23, 24, 25, 26, 27, 28, 29, 30, 31, 32, 33]), Table 2 (Ref. [20, 27, 28, 34, 35, 36, 37, 38, 39, 40, 41, 42, 43, 44, 45, 46, 47, 48, 49, 50, 51, 52]).

**Table 1.** **Risk factors for development of pulmonary hypertension in connective tissue diseases**.

Systemic sclerosis	Antibodies: anticentromere, U3-RNP, anti-Ro52-kDa/SSA, and anti-Th/To [5, 6, 7, 8]
	Disease manifestations: calcinosis, telangiectasias, severe Raynaud’s, or abnormal nailfold capillaries [5, 6, 9, 10, 11]
	Clinical features: female gender, older age at diagnosis, postmenopausal status, lower DLCO [12, 13, 14]
Systemic lupus erythematosus	Antibodies: SSA, SSB, Smith, U1-RNP [15, 16, 17]
	Disease manifestations: antiphospholipid antibody syndrome (aCL, β2GP, LAC), serositis, ILD, RP [15, 16, 17, 18]
	May not correlate with disease activity index [19]
Sjogren’s syndrome	Antibodies: SSB, U1-RNP [20]
	Disease manifestations: severe sicca symptoms (i.e., positive corneal staining) [20]
	Clinical features: younger age of disease onset [20]
Rheumatoid arthritis	Overlap diseases: CPFE, CTEPH [21, 22]
	Clinical features: leflunomide use [23]
Inflammatory myopathies	Disease manifestations: antisynthetase syndrome, ILD, PVOD, CTEPH [24, 25, 26, 27]
Mixed connective tissue disease	Antibodies: U1-RNP, Smith, SMN complex, aCL, β2GP, LAC [28, 29, 30]
	Disease manifestations: pericarditis, polyarthritis, thrombocytopenia, abnormal nailfold capillaries, ILD [28, 31, 32, 33]

DLCO, diffusing capacity of carbon monoxide; aCL, anticardiolipin; β2GP, beta-2 glycoprotein; LAC, lupus anticoagulant; RP, Raynaud phenomenon; ILD, interstitial lung disease; CPFE, combined pulmonary fibrosis and emphysema; PVOD, pulmonary veno-occlusive disease; CTEPH, chronic thromboembolic pulmonary hypertension; SMN, survival motor neuron; SSA, Sjogren syndrome antigen A; SSB, Sjogren syndrome antigen B; U3-RNP, U3-ribonucleoprotein.

**Table 2.** **Risk factors for increased mortality pulmonary hypertension associated with CTD according to etiology**.

Systemic sclerosis	Combined pre- and post-capillary PH [34, 35]
	PVOD, ILD, COPD, or CPFE [34, 36, 37]
	Left ventricular dysfunction [35]
	Late diagnosis, male sex, African-American race, higher PVR [38, 39, 40, 41]
Systemic lupus erythematosus (SLE)	Worse functional class, higher mean PA pressure, higher PVR [42]
	Shorter 6MWD and higher BNP [42]
	Lupus nephritis, pleuritis, pericarditis, ILD, left ventricular dysfunction [43, 44]
	Antiphospholipid antibody syndrome, especially with CTEPH [43, 45, 46]
Sjogren’s syndrome	Low cardiac index, high PVR, longer time to diagnosis [20, 47]
	High Sjogren’s disease damage index [20]
Rheumatoid arthritis	Unknown
Inflammatory myopathies	ILD, particularly rapidly progressive [27, 48, 49]
	High PVR, severe RV dysfunction [50]
Mixed connective tissue disease	Raynaud’s, livedo reticularis [51]
	Tobacco exposure [28]
	Delayed diagnosis and treatment [52]

PH, pulmonary hypertension; PVOD, pulmonary veno-occlusive disease; ILD, interstitial lung disease, COPD, chronic obstructive pulmonary disease; CPFE, combined pulmonary fibrosis and emphysema; PA, pulmonary artery; PVR, pulmonary vascular resistance, CTEPH, chronic thromboembolic pulmonary hypertension; BNP, B-type natriuretic peptide; 6MWD, 6 minute walk distance; CTD, connective tissue disease; RV, right ventricular.

## 2. Systemic Sclerosis 

Systemic sclerosis (SSc) is a rare and debilitating autoimmune CTD characterized by immune dysregulation and progressive fibrosis of skin, internal organs and vasculature. PH is a feared complication of the disease due to devastating morbidity and mortality outcomes. Despite only affecting approximately 10–15% of patients, it is the leading cause of death in SSc, with a 5-year survival as low as 25% [2, 53]. Thus, development of PH is a major risk factor for morbidity and mortality in SSc.

Risk factors for development of PH in SSc are not entirely consistent between studies, but some patterns exist. Limited cutaneous systemic sclerosis (lcSSc) has traditionally been considered a risk factor for PH in SSc, but more recent data suggests a similar prevalence of PH in both diffuse and limited disease subtypes [9, 54]. Antibodies implicated as possible predictive markers for PH in SSc include anticentromere (typical of limited cutaneous disease), U3-ribonucleoprotein (U3-RNP) antibodies, anti-Ro52-kDa/Sjogren syndrome-related antigen A (SSA) antibodies, and anti-Th/To antibodies [5, 6, 7, 8]. SSc-specific risk factors include the presence of gastroesophageal reflux, calcinosis, mild interstitial lung disease (ILD) and more severe vasculopathy as manifested by telangiectasias, digital ulcers, severe Raynaud’s, and abnormal nailfold capillaries [5, 6, 9, 10, 11]. Demographic risk factors include female gender, older age at diagnosis and postmenopausal status, arguing for a possible protective role of estrogen [12, 13].

PH in SSc is increasingly understood to be a heterogeneous clinical entity with overlapping phenotypes. Classically, SSc has been associated with PAH resulting from fibrotic vasculopathy, as well as PH secondary to ILD. As mortality outcomes in the disease improve, there are increasing cases of PH associated with left heart disease. These phenotypes are not mutually exclusive and frequently overlap to varying degrees, rendering the use of traditional World Health Organization (WHO) groupings somewhat challenging.

Several aspects of SSc-PAH which contribute to worse outcomes are worth discussing in detail. As compared to patients with idiopathic PAH, patients with SSc-PAH have poorer survival despite better baseline hemodynamics on right heart catheterization, with lower PVR and mean pulmonary artery pressure (mPAP) [53]. SSc-PAH is also a disease which is less responsive to PAH therapy [55] and compensatory right ventricular (RV) remodeling does not occur as robustly in patients with SSc-PAH and does not recover as well with PAH therapies as it does in idiopathic PAH [56, 57]. Since RV function is a major driver of PAH morbidity and mortality, it is likely this inability to adequately compensate for PVR, both at rest and with exertion, that substantially contributes to the poorer prognosis of these patients. Nonetheless, subgroup analyses of major trials of PAH therapies that examined CTD-PAH, most of which included predominantly SSc-PAH, as well as several studies looking specifically at SSc-PAH, have shown that PAH therapy is effective in improving symptoms, 6 minute walk distance (6MWD), and PVR [58]. Finally, a subset of SSc patients (possibly around 7% of SSC-PAH) also develop PH associated with pulmonary veno-occlusive disease [34, 36], which is seen more commonly in patients with SSc-PAH than in patients with SSc without PAH or ILD [59]. Patients with a greater number of radiological features on computed tomography (CT) consistent with pulmonary veno-occlusive disease (PVOD) have a substantially higher mortality [34]. As patients with PVOD are unable to tolerate PAH therapy well, it is unsurprising that this group has higher mortality [59].

Older age is a known risk factor for developing PH in SSc, but it is also accompanied by increased incidence of left heart disease, which may often be subclinical. In fact, a substantial number of patients with SSc have abnormal deposition of myocardial collagen despite the absence of clinical heart failure [60], and myocardial fibrosis detected by cardiac magnetic resonance imaging is associated with major adverse cardiac events in SSc [61]. Patients with SSc who develop combined PH and left ventricular dysfunction have a substantially lower survival as compared to patients without either, and still lower survival as compared to patients with SSc who have one of the two [34, 35]. Patients at particular risk are those who have combined pre- and post-capillary PH [34].

There is a substantial overlap of patients with SSc who develop ILD and PH. This group is known to have lower survival and quality of life as compared to patients with SSc and isolated PAH [37]. Unfortunately, PH is diagnosed late in patients who have SSc ILD, and echocardiography, the most common detection tool, has a low negative predictive value and often provides an inaccurate estimate of PA systolic pressure, owing in part to difficulty visualizing the heart through fibrotic parenchyma [62]. Higher mortality is also observed in patients with PH and ILD who have PVR ≥8 Wood units, but not higher mean PA pressure, highlighting PVR as the major marker of disease severity in the PH-ILD population [63]. The development of PH among patients with ILD is not predicted by severity of ILD [64]. This highlights the complex relationship between PH and ILD, which in some disease states (such as SSc), may represent a spectrum of phenotypic expressions of pulmonary vascular or parenchymal fibrosis [65]. Although this raises the exciting possibility that PAH-specific therapy may be effective in some patients with PH associated with ILD, only inhaled treprostinil has been successfully demonstrated to have a meaningful benefit for patients with PH associated with ILD [66]. Finally, patients with SSc and PAH who have coexisting chronic obstructive pulmonary disease (COPD) or combined pulmonary fibrosis and emphysema (CPFE) are at highest risk for mortality; patients with CPFE and SSc-PAH have a 2-year survival of under 50% [34].

These overlapping phenotypes are particularly meaningful for mortality outcomes. In a large German registry of 3257 patients with SSc followed for 5 years, patients with ILD and PH (i.e., ILD-PH) had worse 5 year survival (79%) compared to those with PAH alone (85%) [67]. The mortality difference held true even after multivariate analysis, controlling for age, sex, body mass index, and diffusing capacity of carbon monoxide (DLCO). Patients with SSc who have overlapping PAH, left heart disease, and ILD or COPD are at particularly high risk of poor survival [34].

As might be expected, late diagnosis and diagnosis at the time of severe disease portends a poor prognosis for patients with SSc and PH. A lower DLCO, higher pulmonary artery pressures (PAP), higher PVR or worse functional class at the time of diagnosis are independent predictors of mortality in multiple studies [14, 38, 39, 40]. Early diagnosis is admittedly difficult in this population due to the nonspecific nature of symptoms, but regular screening incorporated into clinical practice has improved survival outcomes [68]. Furthermore, use of multimodal screening, such as use of the DETECT algorithm, may be superior to echocardiography alone in identifying patients with PH in SSc [69].

In the United States, although it is uncertain whether African Americans with SSc are more likely to develop PAH, those who do tend to present with more severe disease, as demonstrated by worse functional class, higher B-type natriuretic peptide (BNP), a lower cardiac index, higher PVR, and worse RV function, all corresponding to worse survival, as compared to Americans of European descent [41]. These differences may be related in part to biologic factors, such as differences in RV structure between African Americans and Americans of European descent [70], and in part to socioeconomic differences in access to appropriate care faced by African American patients.

The relationship between sex and mortality in SSc-PAH remains uncertain. Although SSc-PAH is much more common in women compared to men, men may have higher mortality from the disease [38], though there is conflicting evidence regarding this [71]. Additionally, there appears to be a shorter time from SSc diagnosis to PAH diagnosis in men [71], potentially reflective of more severe disease at baseline or gender differences in health behaviors (i.e., willingness to seek medical care) which may lead to delayed presentations [72].

In summary, information gleaned from reasonably large registry studies for SSc suggest that PH-ILD, late diagnosis with worse hemodynamics at time of diagnosis, male gender, African-American race, and lack of appropriate combination therapy up-front may confer increased mortality risk for patients with PH-SSc. As we expect continued improvement in overall mortality outcomes in the SSc population with the advent of newer therapies, the prevalence of SSc-PH will likely increase—further stressing the importance of multimodal screening, appropriate diagnosis with right heart catheterization, and multi-drug treatment.

## 3. Systemic Lupus Erythematosus 

Systemic lupus erythematosus (SLE) is a complex autoimmune disease characterized by the production of autoantibodies, immune complex formation and deposition and other immune abnormalities with multi-organ dysfunction. Though pulmonary disease is common in patients with SLE, pulmonary vascular disease is a rare manifestation. SLE has the second highest prevalence of PH after SSc amongst autoimmune diseases, with most cases of PH presenting within the first five years of SLE diagnosis and a female sex predominance [73]. The overall incidence of PH is somewhere between 1–10% of patients with SLE [73, 74, 75, 76, 77]. When looking at registry data of PH patients, SLE is the second most common cause of CTD-PAH after SSc in Europe; however, in China, Taiwan and Korea, SLE may be at least as common, if not more so, than SSc as cause for PAH [2, 77]. Patients may be more likely to develop PAH in SLE if they have lupus anticoagulant (LA) or positive SSA, Sjogren syndrome antigen B (SSB), Smith, and U1-RNP antibodies [15, 16, 17]. Although the development of serositis, ILD, and Raynaud phenomenon may increase the risk of PAH in SLE, in general disease activity does not seem to correlate to likelihood of PAH incidence [15, 17, 19]. As compared to SSc-PAH, SLE-PAH seems to have a more favorable prognosis, with a 5-year survival of around 68–85% [15, 42]. The development of PAH in SLE may be associated with higher mortality [78].

The pathogenesis of PAH in SLE is likely related to a complex interaction of immune-mediated inflammation of pulmonary parenchyma and vasculature and microthrombosis leading to pulmonary vascular remodeling [79]. Deposition of antinuclear antibodies, anti-dsDNA, immunoglobulins, and complement in the pulmonary arterial walls appear similar to histologic findings in lupus nephritis and suggest that pulmonary vasculitis may be a significant driving factor for disease [79, 80]. These observations may help to explain the benefit seen in largely uncontrolled studies of immunosuppression for SLE-PAH; these small studies, most of which used a combination of cyclophosphamide and prednisone, suggest an improvement in pulmonary hemodynamics [79]. However, it is uncertain whether this truly represents a benefit of immunosuppression, since many of the patients enrolled in these studies also received PAH-directed therapy.

Among patients who develop PAH in SLE, those who have a worse functional class, higher mean PA pressure, higher PVR, lower 6MWD and higher BNP are at higher risk for mortality [42]. Renal involvement in SLE is an additional risk factor for mortality among patients with SLE-PAH [15, 78]. Interestingly, the presence of U1-RNP antibodies may be protective for survival [15], though with conflicting evidence [43]. Since U1-RNP positivity is a known risk factor for PAH development, it may be that patients who are U1-RNP positive undergo diagnostic testing and treatment for PAH earlier in the course of disease, depending on local practice patterns. As in patients with PAH in general, patients with SLE-PAH who respond well to pulmonary vasodilator therapy (i.e., achieving New York Heart Association (NYHA) functional class I-II, normalization of BNP and RV function, and increase in 6MWD) have a better prognosis than those who do not [81]. The management of SLE-PAH is the same as for all WSPH group 1 PAH, and limited subgroup analysis of SLE patients in PAH trials suggests that these patients are likely to experience a comparable benefit to patients with idiopathic PAH [58].

As in other CTDs, the development of overlap syndromes in SLE-PH can contribute to higher mortality. Coexisting cerebrovascular disease, pleuritis or pericarditis, and ILD are a significant risk factor for mortality in patients who have PH in SLE [43]. The presence of antiphospholipid antibody syndrome (APLS) seems to increase the risk of PAH and associated mortality, although this is not a universal observation [18]. However, since CTEPH occurs frequently in APLS, patients with SLE who have positive anticardiolipin, beta-2 glycoprotein, and especially LA (particularly with multiple positivity) are likely at increased risk for mortality and should be closely monitored for CTEPH development [45], an observation which seems to be particularly notable in CTD-associated APLS [46]. Patients with CTEPH associated with APLS should be considered for pulmonary thromboendarterectomy (PTE) in the same manner as those without APLS, but patients with APLS appear to be at higher risk of postoperative stroke, thrombocytopenia, and delirium [82]. Certain APLS-specific features, such as higher titers of antiphospholipid antibody IgG, may be associated with postoperative neurological impairment [83]. In experienced centers with appropriate planning, mortality and reduction in PA pressure and PVR after PTE for APLS-associated CTEPH is comparable to that of patients without APLS [45, 82, 84]. However, patients with APLS-associated CTEPH may have higher rates of recurrent postoperative CTEPH and need for repeat PTE [85].

SLE can be associated with left ventricular systolic dysfunction, but APLS also seems to increase the risk of biventricular diastolic dysfunction, which can contribute to an overlap PH syndrome in some patients [44]. Patients with APLS, with and without SLE, should be considered for indefinite anticoagulation, particularly if they have had prior thromboembolic events.

In summary, patients with SLE may be at increased risk for PH if they have particular antibody patterns or APLS. The development of PAH seems to impact survival in SLE, and although no PH screening guidelines exist in SLE, clinicians should consider the possibility of this diagnosis, refer for right heart catheterization early, and start aggressive multimodal therapy as for other forms of PAH.

## 4. Sjogren Syndrome

Primary Sjogren’s syndrome (pSS) is an autoimmune disorder characterized by lymphocytic infiltration of exocrine glands with resultant sicca symptoms. It is not typically considered a major risk factor for PH; however, newer data suggests that these patients may be at an increased risk of morbidity and mortality due to PH.

The incidence of PH in patients with pSS is poorly understood. The French health insurance database study suggests that patients with pSS are significantly more likely to be hospitalized for PH compared to age and sex matched hospital inpatients [86]. Several small-scale studies and case series, some of which use echocardiographic definition for PH, also suggest a higher prevalence of PH in patients with pSS [87, 88]. Among patients enrolled into PH registries, the prevalence of pSS-PAH ranges from under 1% to as high as 15% [77, 89, 90].

Sjogren’s specific risk factors for PH include younger age of disease onset, severe sicca symptoms (including positive corneal staining), and specific antibodies including anti-SSB and anti-U1-RNP [20]. In whole exome sequencing of 34 patients with pSS-PAH, 141 pathogenic variant loci of 129 genes were identified [91].

As little is known about true incidence of PH as well as risk factors thereof for the pSS population, there is even less known about predictors of morbidity and mortality outcomes. One particular multicenter cohort study from China demonstrated that low cardiac index, high pulmonary vascular resistance as well as high Sjogren’s disease damage index were associated with increased risk of death in 103 patients with pSS-PAH [20]. In another Chinese study of 29 patients with right heart catheterization (RHC)-confirmed pSS-PAH, shorter time from pSS to PAH diagnosis, higher cardiac index, and use of immunosuppressive medications were associated with improved survival suggesting mortality benefit in early diagnosis of PAH and immunosuppression for pSS [47].

In summary, though not robust, data suggests that pSS are particularly vulnerable to morbidity and mortality outcomes related to PH which may be mitigated by early diagnosis and immunosuppression.

## 5. Rheumatoid Arthritis

Rheumatoid arthritis (RA) is an autoimmune disease which typically affects women between the ages of 40–60 years old leading to progressive erosive arthropathy requiring treatment with immunosuppression. Though rarer, extra-articular manifestations are not uncommon. Pulmonary manifestations include ILD, pleural effusions, pulmonary nodules, small airway disease and PH [92].

The data on the association of rheumatoid arthritis and PH, as well as contributors to adverse outcomes, are limited; echocardiographic studies have suggested a moderately high prevalence of PH in patients with RA (around 14–20%), but these have been limited by low frequencies of confirmatory right heart catheterization [93, 94]. Patients with RA have also been found to have a lower ratio of tricuspid annulus plane systolic excursion (TAPSE) to RV systolic pressure, a marker of RV dysfunction, as compared to patients without RA [95]. This risk, as well as that of cardiovascular disease in general, may be higher for patients with seropositive RA [96]. Overall, these data suggest that although RA is not as common a driver for PAH as other CTD [97], PH is probably underdiagnosed in patients with RA. As compared to patients with idiopathic PAH, patients with RA-PAH are diagnosed at an older age and may have slightly lower mean PA pressure; mortality outcomes are comparable, but these are challenging to interpret because of the relative rarity of the disease [98].

Patients with RA who are smokers seem to have an increased likelihood of CPFE, which is associated with increased PH risk [21, 22]. Leflunomide, used commonly in RA, seems to be associated with a risk of PH [23], and it is uncertain whether the studies mentioned earlier demonstrate a PH risk from RA or from leflunomide. Finally, RA is a risk factor for pulmonary embolism [99], which suggests the need for monitoring of these patients for CTEPH.

RA-PH requires further research to understand whether there is a true association between the disease states, and if so, what the risk factors are for development of PH in RA and the risk factors for morbidity and mortality in this population.

## 6. Inflammatory Myopathy

Inflammatory myopathies (IM), which include polymyositis (PM), dermatomyositis (DM), and antisynthetase syndrome (AS), are a subgroup of CTDs defined by autoimmune muscle injury. The amyopathic variant of DM is defined by skin changes without muscle involvement. A significant proportion of patients with IM develop ILD, which is a major driver of mortality and primary cause of death in this population [100].

Less established is the incidence of PH in patients with IM. Epidemiologic data in polymyositis and dermatomyositis have estimated the incidence of PH between 6–60%; however, these data are predominantly obtained by echocardiography and are often not confirmed by right heart catheterization [24]. In PH registries where such granularity is available, IM-PH accounts for about 4% of cases [2]. Most cases of PH seem to be associated with ILD or pulmonary embolism; in the largest well-characterized population of patients with PH, the incidence of precapillary PH without ILD was 0.06% [101]. ILD is more common among patients with AS, particularly in patients who have antibodies against aminoacyl transfer RNA synthetasese (such as anti-Jo-1), as well as in patients with IM who are have positive anti-Ro52 and anti-melanoma differentiation-associated protein 5 (anti-MDA-5) antibodies [25]. Thus, it might be expected that these patients also are higher risk of PH. When present, PH dramatically and independently impacts prognosis of patients with IM-ILD; for example, the survival of AS-associated ILD with PH is far lower than that of ILD alone [27].

Since little is known about PH in IM outside of ILD, the tempo of ILD progression is a major risk factor for mortality. About 9% of patients with IM develop rapidly progressive ILD, with a remission rate of around 50–60% when treated with aggressive immunosuppressive therapy [48, 49]. The high incidence and significant impact of ILD on the course of patients with IM highlights the need for early diagnosis, close monitoring, and aggressive treatment of these patients. Since the PH present in these patients is often WSPH group 3 disease, they merit treatment with inhaled treprostinil; however, among patients with high PVR or significant RV dysfunction, treatment with systemic pulmonary vasodilators, including parenteral prostacyclin therapy, can be considered [50].

Additional causative factors for PH in IM include pulmonary embolism and pulmonary veno-occlusive disease, both of which have rarely been reported [24, 26]. Finally, the risk of malignancy in DM and PM can contribute to mortality risk in IM; however, AS and ILD are associated with a somewhat lower cancer risk, so malignancy may not be as much of a risk factor among patients who have PH [102].

## 7. Mixed Connective Tissue Disease

Mixed connective tissue disease (MCTD) is a complex clinical entity defined by the presence of overlapping features of SLE, SSc, myositis and the presence of anti-U1-RNP antibodies. As expected, diagnosis of this condition is especially challenging given its heterogeneous nature and wide spectrum of disease. In fact, there is controversy regarding whether this is truly a distinct disease process or a term utilized for patients with undifferentiated CTD [103]. The limited data we have suggests that these patients may be at increased risk for PH and PH related morbidity and mortality.

Owing to regional and individual differences in clinical practice and diagnostic style, there is enormous inter-study variability in patient characteristics. Although it is suspected that patients described as having MCTD likely do have a notable incidence of PH, there is very little consistent data on the matter. Most studies which have attempted to assess the prevalence of PH in MCTD patients have been limited by use of transthoracic echocardiography (TTE) instead of right heart catheterization. One study out of Norway demonstrated a 3.4% prevalence of PH in their 147 patient cohort and another from Taiwan demonstrated a 0.33% prevalence in 1811 patients with myositis, whereas a smaller study of 26 MCTD patients in Romania demonstrated 46.15% prevalence of PH [31, 77, 104]. The true prevalence may be in between, as a meta-analysis of 8 studies with 983 pooled patients found a prevalence of 12.53% [105], suggesting that these patients may truly be at risk for PH. Interestingly, MCTD-PAH and SLE-PAH are the leading causes of CTD-PAH in Asian countries, in contrast to SSc which is the leading cause of CTD-PAH in Western countries, thus introducing an additional confounding variable [106].

A wide range of risk factors have been associated with the development of PH in MCTD. These include pericarditis, polyarthritis, thrombocytopenia, ILD, as well as the presence of certain antibodies (i.e., anti-Sm, anti-survival motor neuron (SMN) complex, anticardiolipin (aCL), anti-beta-2 glycoprotein I (aβ2GPI) antibodies and LAC) [28, 29, 31]. Anti-U1-RNP antibodies, which are necessary for diagnosis of MCTD, but are not pathognomonic for it, have been identified as protective for survival in CTD and SSc associated PAH [30]. Interestingly, several studies suggest that abnormal nailfold capillaries, and nailfold capillary patterns similar to that of SSc patients predicts development of PH in patients with MCTD, thus lending itself as a useful tool for risk stratification and early diagnosis of PH [31, 32, 33]. These findings suggest a common underlying vasculopathy which explains the development of Raynaud’s and abnormal nailfold capillaries, as well as the development of MCTD-PH.

Ultimately, the relevance of these MCTD-PH risk factors is to identify patients who may be at increased risk of mortality. Common to the theme of PH in CTD, patients with MCTD-PH tend to have mortality driven by PH, with an approximately 56% 10-year mortality in one study [28].

Mitigating the underlying vasculopathy may be critical to improving outcomes in patients with MCTD-PH. When attempting to cluster MCTD patients according to disease phenotype, those with the presence of Raynaud’s, livedo reticularis, and PAH tended to have the worst prognosis [51]. The MCTD-PAH study of the French Registry demonstrated that tobacco exposure is an independent risk factor for MCTD-PAH mortality [28]. This is a very modifiable risk factor which may also serve to give agency to this patient population.

Early diagnosis and management is likely to improve disease outcomes. Systemic pulmonary vasodilators used in other types of PAH have been found to be well tolerated in this population as well. A study of macitentan demonstrated similar safety and treatment outcomes in patients with CTD-PAH (including MCTD-PAH) as in those with idiopathic PAH; however, MCTD patients the most advanced disease at time of drug initiation as compared to other CTD, and subsequently, had the highest proportion of hospitalizations and likelihood of treatment escalation [52]. As compared to SSc-PH, MCTD-PH may be more responsive to immunosuppression, with older studies demonstrating improvement in functional classification and PA pressure with systemic corticosteroids or cyclophosphamide [107, 108].

MCTD is a nebulous disease with a wide range of phenotypes, but evidence does suggest that patients may be at risk for PH and subsequent complications, including death. The supporting data is sparse with heterogeneous patient populations, rendering risk analysis for morbidity and mortality very difficult.

## 8. Future Directions

The common thread of delayed diagnosis and presentation at advanced stages of disease in CTD-PAH has raised interest in novel technologies, including artificial intelligence (AI), to characterize PH phenotypes and outcomes and, potentially, to rapidly screen large populations for early disease diagnosis. Specifically, AI algorithms have been demonstrated to reliably predict PH using electrocardiograms [109], to diagnose PH and predict mortality using echocardiography [110, 111], and to assess parenchymal lung disease in PH [112]. If well validated and broadly applied, these tools combined with molecular diagnostics have the potential to identify patients who require urgent evaluation and phenotype PH in various CTDs to predicted an optimal treatment regimen.

The presence of overlap syndromes with left heart disease, parenchymal lung disease, and persistent, long-term inflammation, all of which are risk factors for poor outcomes in CTD-PAH, highlights the need to consider the addition of medical therapy past PH-specific agents. The advent of sodium-glucose contransporter 2 inhibitors (SGLT2i), glucagon-like peptide-1 (GLP1) agonists, and angiotensin receptor/neprilysin inhibitors (ARNI) in cardiovascular disease has raised the exciting potential for these therapies in PAH [113]. Although the most obvious application of these is in patients who have overlap syndromes with left heart disease, a common and significant risk factor in CTD, the impact of these three drug classes on reduction in RV remodeling [114, 115], myocardial inflammation associated with metabolic dysfunction [114, 116], and PA pressure [117, 118, 119], as well as potential benefits on slowing of progression of renal failure, a known risk factor for worse outcomes in PAH [120], suggests that future studies may show benefits unique to patients with different subtypes of CTD. Novel approaches to drug discovery may provide avenues to potential candidate therapies specific to CTD-PH [121].

Importantly, it is critical to include a future research focus on patients at high risk for CTD-PAH who have poor access to advanced medical care, such as those living in developing countries. Epidemiologic data on incidence, phenotypes, and outcomes is limited in those populations and often contrasts with that which is reported in Europe, Canada, and the United States [77, 122, 123], and patients are often unable to obtain accurate diagnostics or guideline-directed treatment in a timely fashion, which likely contributes to poorer outcomes [76, 123, 124, 125].

## 9. Conclusions

CTD-PH is a highly complex, heterogeneous condition which requires clinician attentiveness to the unique features of each patient’s disease. Although CTD-PAH is generally treated with the same systemic pulmonary vasodilator therapy as other patients in WSPH group 1, these patients often have worse outcomes despite a more favorable hemodynamic profile. Furthermore, in some CTDs, the diagnosis of PH arrives only at advanced stages of the disease, making these patients potentially less responsive to therapy. Finally, a significant amount of disease overlap exists in this population, highlighting the need for meticulous diagnostics and close monitoring. A large research gap exists in many CTDs when considering PH, and future work should focus on these patients’ unique characteristics and clinical trajectories. Finally, research and clinical work in CTD-PAH should be inclusive of patient populations across the world, particularly in populations with poorer access to diagnostics and treatment options.
